# Supplementary material for: Exploring the Anticancer Potential of Phenolic nor-Triterpenes from Celastraceae Species
Source: Int J Mol Sci. 2024 Aug 30;25(17):9470. doi: 10.3390/ijms25179470 (PMC11395069; doi:10.3390/ijms25179470)
Supplement: Supplementary file 1 [file ijms-25-09470-s001.zip › ijms-3143943-supplementary.pdf]

Supplementary Materials

Exploring the anticancer potential of phenolic *nor*-triterpenes from Celastraceae species

Carolina P. Reyes[1], Alejandro Ardiles[2], Laura Anaissi-Afonso[3], Aday González-Bakker[4], José M. Padrón[4], Ignacio A. Jiménez[4], Félix Machín[3,5,6,*], Isabel L. Bazzocchi[4,*]

1 Instituto Universitario de Bio-Orgánica Antonio González and Departamento de Bioquímica. Microbiología, Biología Celular y Genética, Universidad de La Laguna, Av. Astrofísico Francisco Sánchez 2, 38206 La Laguna, Spain; cpreyes@ull.edu.es.
2 Universidad Santo Tomás, Departamento de Ciencias Básicas, facultad de Ciencias, Avenida Iquique 3991, Antofagasta, Chile; aardiles2@santotomas.cl.
3 Unidad de Investigación, Hospital Universitario Ntra Sra de Candelaria, Ctra del Rosario 145, 38010 Santa Cruz de Tenerife, Spain; anaissi.ull@gmail.com.
4 Instituto Universitario de Bio-Orgánica Antonio González and Departamento de Química Orgánica, Universidad de La Laguna, Av. Astrofísico Francisco Sánchez 2, 38206 La Laguna, Spain; agonzaba@ull.es; jmpadron@ull.es; ignadiaz@ull.edu.es.
5 Instituto de Tecnologías Biomédicas, Universidad de La Laguna, 38200 La Laguna, Tenerife, Spain.
6 Facultad de Ciencias de la Salud, Universidad Fernando Pessoa Canarias, 35450 Las Palmas de Gran Canaria, Spain.

Table of Contents

Figures S1-S3. ^{1}H and ^{13}C NMR, and MS spectra of novel metabolites (**1**-**3**) isolated from *Celastraceae species*.

Table S1. Minimum inhibitory concentration (MIC) of phenolic nor-triterpenes against selected bacterial strains.

Videos S1-S3: Continuous live cell imaging of HeLa cells exposed to compounds **9**, **12** and **14**.

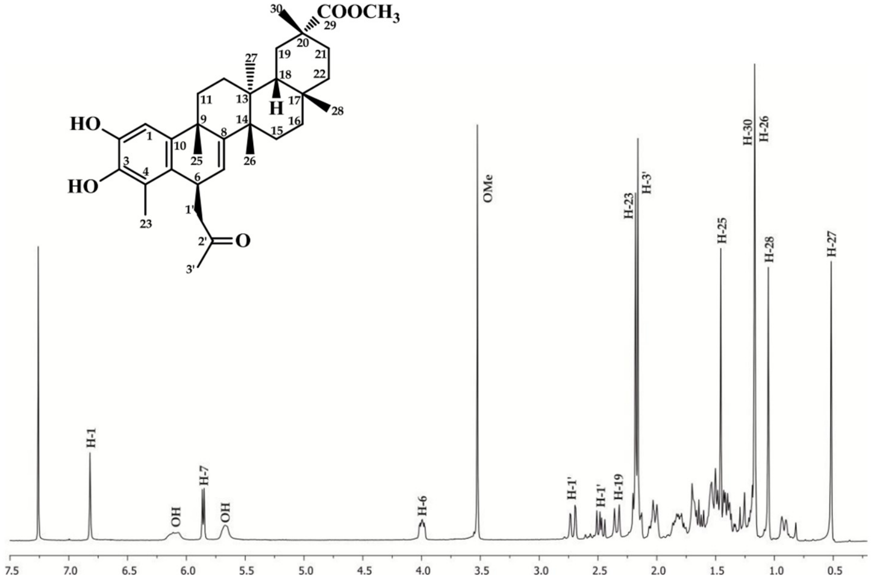

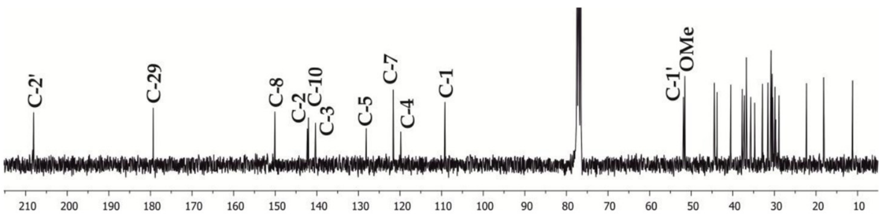

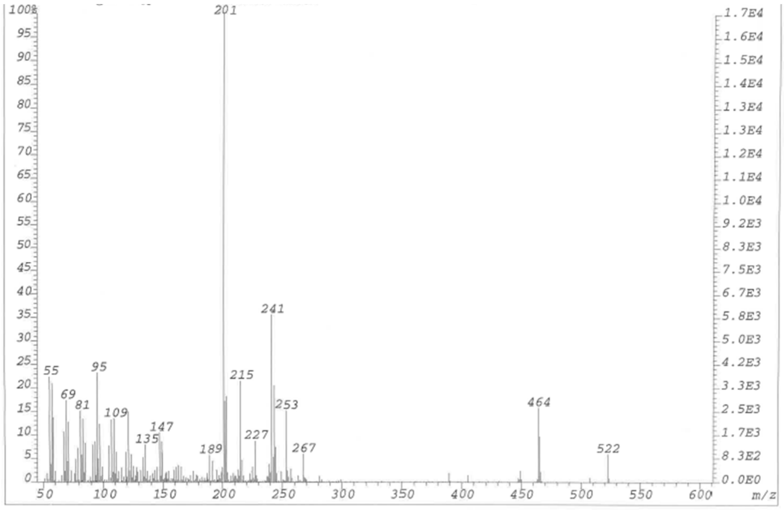


```
AutoSpec EI+ Magnet BpM:201 BpI:19552 TIC:158970 Flags:ACC
File Text:              (              )  EI+ (70 eV) Temp. fuente+ 230 C
  Heteroatom Max:   20    Ion:  Both Even and Odd
  Limits:

  200.000     0.0                                   -0.5    0    0    0
  525.000   100.0                10.0               50.0   33   48    5

    Mass      %RA Pks   Std    PPM   mDa   Calc. Mass    DBE    C    H    O

  523.328033  0.2
  522.336288  5.5               -3.4  -1.8  522.334525   11.0   33   46    5
```

Figure S1. ^{1}H and ^{13}C NMR spectra ($CDCl_3$, 500 and 125 MHz, respectively), and HREIMS spectrum of compound **1**.

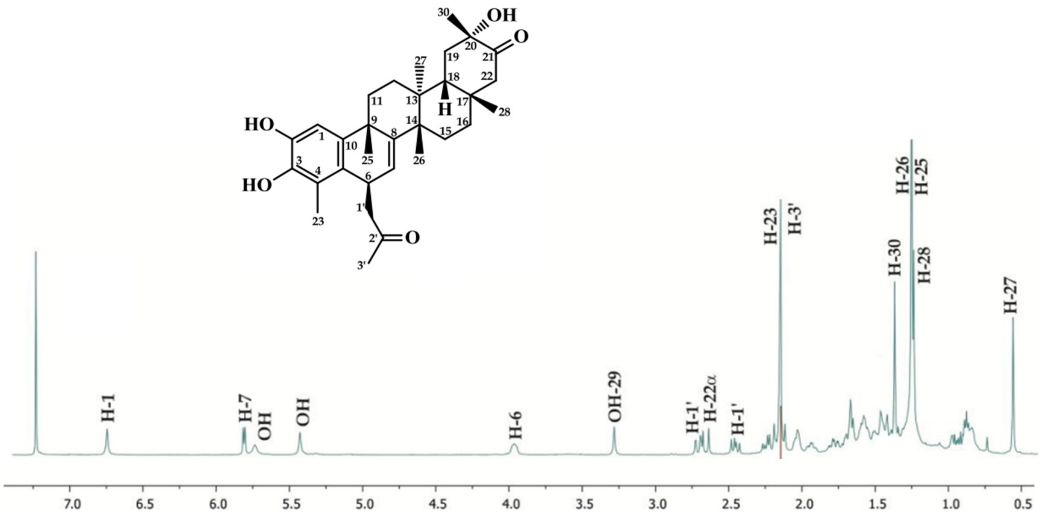

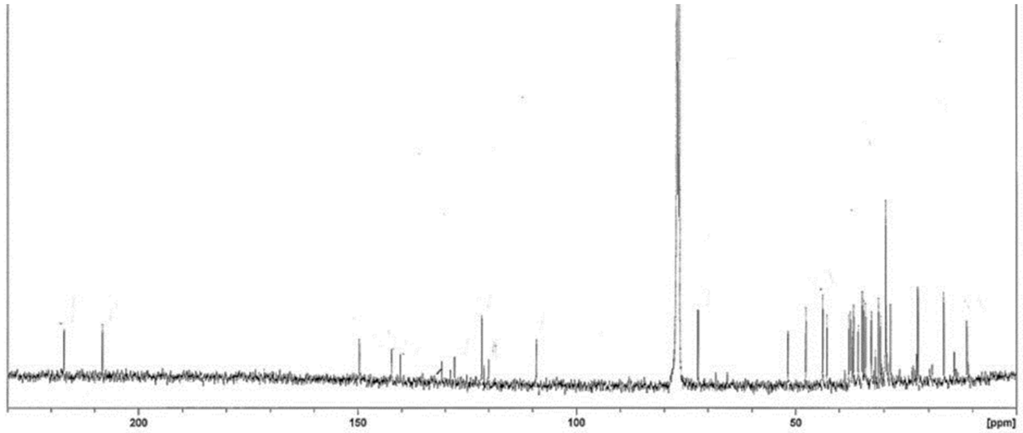

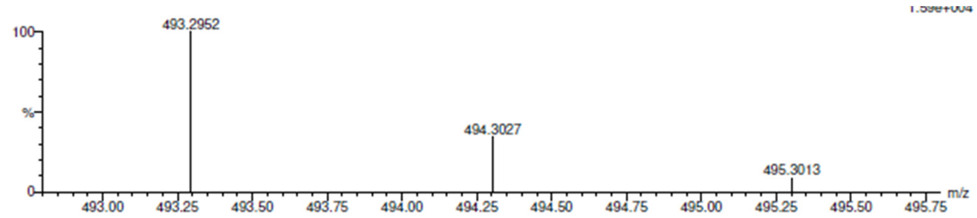

Minimum:	20.00				-1.5			
Maximum:	100.00		5.0	5.0	200.0			
Mass	RA	Calc. Mass	mDa	PPM	DBE	i-FIT	i-FIT (Norm)	Formula
493.2952	100.00	493.2954	-0.2	-0.4	11.5	18.6	0.2	C31 H41 O5
		493.2930	2.2	4.5	8.5	20.3	1.9	C29 H42 O5 Na
494.3027	34.03	---						

Figure S2. ^{1}H and ^{13}C NMR spectra ($CDCl_3$, 500 and 125 MHz, respectively), and HREIMS spectrum of compound **4**.

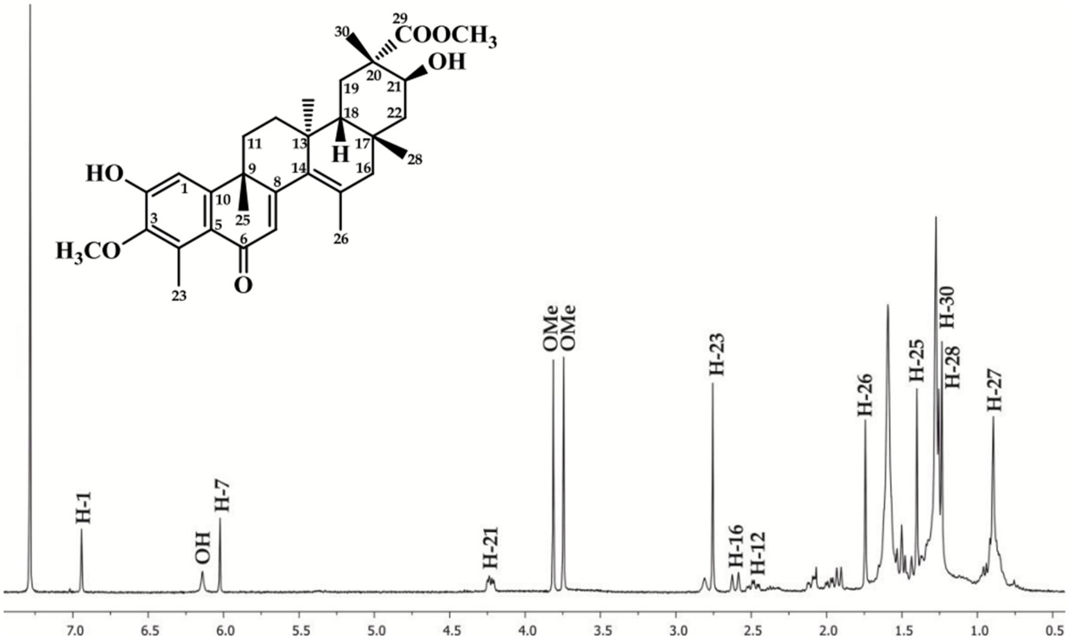

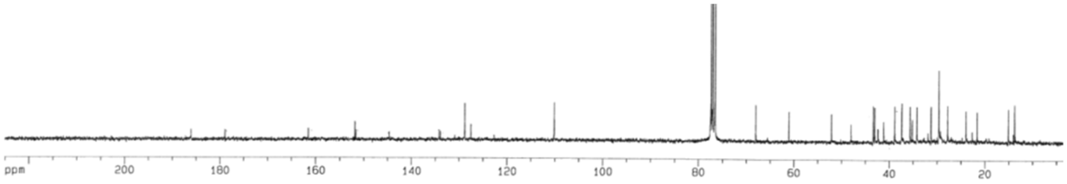

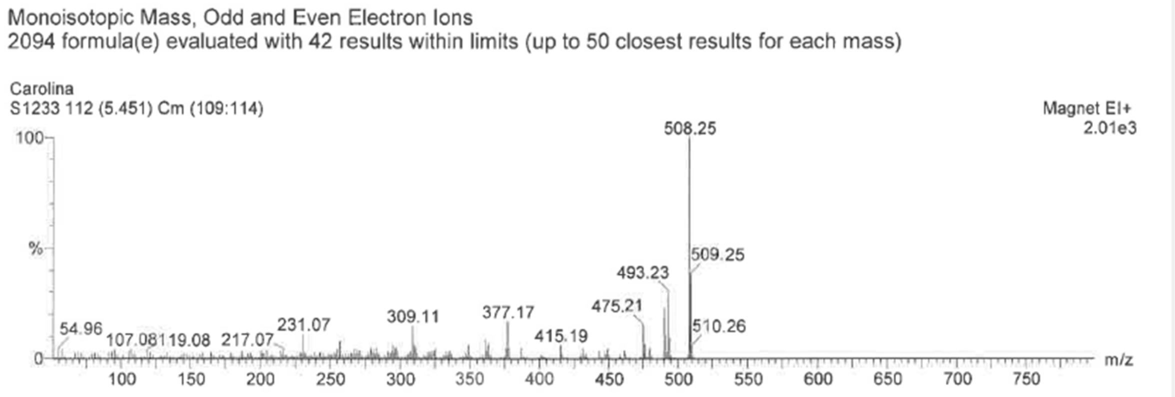

Minimum:	8.00				-1.5	
Maximum:	100.00		200.0	10.0	50.0	
Mass	RA	Calc. Mass	mDa	PPM	DBE	Formula
508.2855	64.44	508.2825	3.0	5.9	12.0	C31 H40 O6
508.2808	70.07	508.2825	-1.7	-3.3	12.0	C31 H40 O6

Figure S3. ^{1}H and ^{13}C NMR spectra ($CDCl_3$, 500 and 125 MHz, respectively), and HREIMS spectrum of compound **6.**

Table S1. Minimum inhibitory concentration (MIC) of phenolic *nor*-triterpenes **1-15** against selected bacterial strains.

**Compound**[s]	***Escheria coli*** (ATCC25923)	***Enterobacter faecalis*** (ATCC29212)	***Staphylococcus aureus*** (NRS402)
**1**	>128	>128	>128
**2**	>128	>128	>128
**3**	>128	>128	>128
**4**	>128	>128	>128
**5**	>128	>128	>128
**6**	>128	>128	>128
**7**	>128	>128	>128
**8**	>128	>128	>128
**9**	>128	>128	>128
**10**	>128	>128	>128
**11**	>128	>128	>128
**12**	>128	>128	>128
**13**	>128	>128	>128
**14**	>128	>128	>128
**15**	>128	>128	>128
Ampicillin	>128	128	>128
Oxacillin	>128	>128	>128
Vancomycin	64	32	64
Mupirocin	16	16	16

[a] MICs for tested compounds are in µM and for reference antibiotics are in mg/L.

Video S1_9.avi

Video S1: Continuous live cell imaging of HeLa cells exposed to compound **9**.

Video S2_12.avi

Video S2: Continuous live cell imaging of HeLa cells exposed to compound **12**.

Video S3_14.avi

Video S3: Continuous live cell imaging of HeLa cells exposed to compound **14.**
